# Appendix P: Guidance for Soil Collection, Characterization, and Application for Biothreat Agent Detection Method and Site Evaluations

**DOI:** 10.1093/jaoacint/qsaa044

**Published:** 2020-06-24

**Authors:** Sharon Brunelle

## 1 Objective

To provide guidance on standardization of practices for the collection, characterization, and use of soil as a sample matrix or potentially interfering substance in biothreat agent detection applications.

## 2 Scope

This standard applies to (1) soils used as a source of potentially interfering substances for the testing and evaluation or validation of biothreat agent detection methods and systems, and (2) soils used as a sample matrix in site assessments and in evaluation of biothreat agent decontamination and remediation procedures.

## 3 Purpose

It is essential to evaluate a candidate biothreat agent detection method or system for inhibition, interference, and cross-reactivity from soil to determine the reliability and/or suitability of the method or system in the presence of this environmental factor. It is also imperative that methods used for site assessments detect reliably the target agent in the soil matrix. Currently, however, there are no generally agreed-upon standards for the preparation and characterization of soils, nor for the use of soil samples in biothreat agent detection applications. This voluntary consensus standard will help to establish uniformity in the use of soils for evaluation of candidate biothreat agent detection methods and field-deployable technologies. This will result in increased confidence in the reliability of methods and systems and allow for direct comparison of data among studies.

## 4 Introduction

Through a voluntary consensus standard development process, the AOAC Stakeholder Panel on Agent Detection Assays (SPADA) has developed 16 *Standard Method Performance Requirements* (SMPRs^®^) for various biothreat agent detection methods (1). As part of the validation requirements, the methods are assessed for environmental interferences, including testing with a variety of soil types. Chemical or biochemical (e.g., nucleic acid or protein) components in soils can cause positive or negative interferences in biothreat agent detection methods and systems based on polymerase chain reaction (PCR) or immunoassay technologies. No guidance, however, is provided in these documents regarding how to choose, collect, process, and test the soils in a standard manner. Guidance is challenging due to the complexity of the operational environment, the impact the various methods and systems under evaluation may have on the type and amount of sample required, and the lack of ability to cover every mission type/constraint that may drive sample choice, collection, and processing.

This standard focuses on two main experimental uses for soils: (1) soils as a positive and/or negative interference in biothreat agent detection methods with a focus on field-deployable detectors and assays during either laboratory or field experiment setups; and (2) soils tested as part of a site survey or as part of pre- and post-decontamination and remediation assessments. In the first instance, soil is not the intended matrix for the method, but soil components may become airborne and be collected on filters, in liquid aerosol collectors, on surfaces, and in water as contaminants. In the second instance, soil is the intended sample matrix for the method.

## 5 Terms and Definitions

The following terms and definitions are from Weil and Brady (2):

- *A horizon*.—Surface horizon of a mineral soil having maximum organic matter accumulation, maximum biological activity, and/or eluviation of materials such as iron and aluminum oxides and silicate clays.
- *B horizon*.—Soil horizon, usually beneath the A horizon, that is characterized by one or more of the following: (1) concentration of silicate clays, iron, and aluminum oxides, and humus, alone or in combination; (2) blocky or prismatic structure; and (3) coatings of iron and aluminum oxides that give darker, stronger, or redder color. The B horizon accumulates clay minerals that have leached from upper layers.
- *C horizon*.—Mineral horizon, generally beneath the solum, that is relatively unaffected by biological activity and pedogenesis and is lacking properties diagnostic of an A or B horizon. The C horizon consists of partially altered parent material and tends to contain more characteristics of the bedrock below or the displaced material deposited at the site.
- *Cation exchange capacity (CEC)*.—Sum total of exchangeable cations that a soil can adsorb. Sometimes called total-exchange capacity, base-exchange capacity, or cation-adsorption capacity. CEC is expressed in centimoles of charge per kilogram (cmolc/kg) of soil (or of other adsorbing material, such as clay).
- *Clay*.—Soil consisting of particles <0.002 mm in diameter. Clay is negatively charged and has capacity for water retention.
- *Clay mineral*.—Naturally occurring inorganic material (usually crystalline) found in soils and other earthy deposits, the particles being of clay size.
- *E horizon*.—Light-colored mineral horizon where most of the organic matter and smaller minerals have eluviated or leached out of the layer.
- *Fine sand*.—Comprised of particles in diameter range of 0.2–0.02 mm. Made of weathered primary rock minerals and particles that do not pack together easily. Air enters easily and water flows through fine sand rapidly.
- *Fulvic acid.—*A term of varied usage but usually referring to the mixture of organic substances remaining in solution upon acidification of a dilute alkali extract from the soil.
- *Humic acid*.—Mixture of variable or indefinite composition of dark organic substances, precipitated upon acidification of a dilute alkali extract from soil.
- *Humin*.—Fraction of the soil organic matter that is not dissolved upon extraction of the soil with dilute alkali.
- *Humus*.—The more or less stable fraction of the soil organic matter remaining after the major portions of added plant and animal residues have decomposed. Usually dark in color.
- *Loam*.—Soil type that contains the basic soil particles sand, silt, and clay, with a smaller amount of clay and a roughly equivalent amount of sand and silt. An example mineral composition of a loam is 40% sand, 40% silt, and 20% clay.
- *O horizon.*—Organic layers of decaying plant and animal tissue containing 12–18% organic carbon excluding the root and large fiber fraction. Oi is a fibric horizon, Oe is Hemic, and Oa is Sapric. This horizon is typically the top horizon in a soil profile when present.
- *Silt*.—Comprising particles in the size diameter range 0.02–0.002 mm. Smaller than sand and more difficult to drain.
- *Soil horizon*.—Layer of soil, approximately parallel to the soil surface, differing in properties and characteristics from adjacent layers below or above it.
- *Soil profile*.—Vertical section of the soil through all its horizons and extending into the parent material.
- *Solum*.—Comprised of surface and subsoil layers that have undergone the same soil-forming conditions.

## 6 Background Information on Soil

There are over 19 000 identified soils in the United States alone, making experimental testing with soils difficult to scope. The study of soil is interdisciplinary involving chemistry, biology, physics, soil genesis, and taxonomy, in addition to agricultural and conservation practices. Soils are an important natural resource. They are a medium for plant growth, a regulator for water supply, a recycler of raw materials, a habitat for soil organisms, an engineering medium, and an environmental interface. Overall, soils are a very complex matrix including physical and living components that lead to ever-changing compositions.

Variability in soils can be problematic. The physical and living components of soil change with depth of the soil, leading to soil horizons in a single soil profile that have different characteristics. With the different characteristics in mind, care must be exercised when collecting samples to avoid mixing soil types. These horizons can have different pH, organic content and clay minerals. Soils also vary seasonally and over time. A collected soil sample is considered a catch sample and represents a snapshot in time of that soil. Outside of the soil profile, soils change with distance such that two soil samples collected only a few feet apart can have very different characteristics. When collecting soil samples, reviewing soil maps and preparing to analyze the sample shortly after collection is recommended in order to confirm the characteristics desired for the experimental purpose. If planning on combining subsamples of collected soil, field texture methods and field soil pH kits are helpful in establishing similar characteristics between the subsamples.

### 6.1 Soil Texture

**Figure 1.**
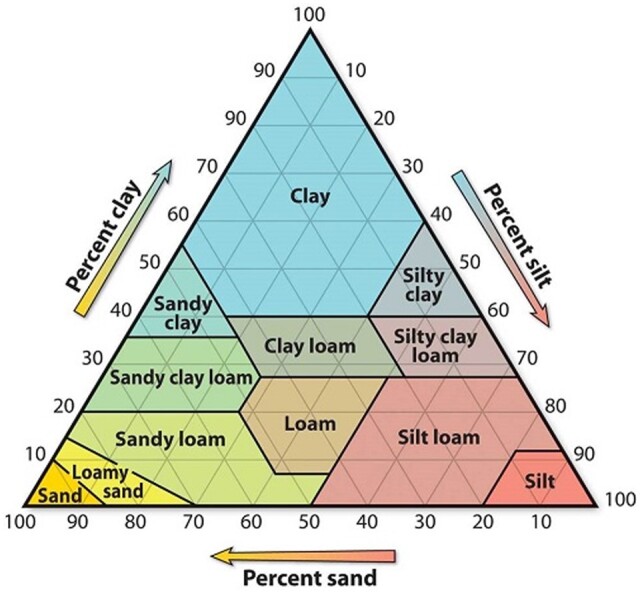
Soil texture triangle. Reprinted from https://soils4teachers.org/files/images/s4t/texture-triangle.jpg

Soil consists of organic and non-organic components. The non-organic components can be divided into three main mineral groups: sand, silt, and clay. Clay is the most active component of soil, having the smallest size and therefore the largest surface area. Any non-organic material >2 mm is considered gravel and is most often not included in soil experiments or testing. The ratio of sand, silt, and clay determines the texture of the soil (Figure 1) and varies little over time. Texture is critical to soil behavior, including gas exchange, active fraction, nutrient retention, and water retention.

### 6.2 Soil Organisms

Soil is also an important habitat for organisms, including microbes, meso fauna (0.1–2 mm), and macro fauna (>2 mm). One gram of soil is typically expected to harbor 10^8^–10^9^ live bacteria. In addition to bacteria and archaea, larger organisms like fungi, protozoa, nematodes, and micro arthropods are found in large numbers.

### 6.3 Soil pH

The pH of a soil impacts the behavior of chemicals and plays a role in the soil ecosystem diversity. The typical pH range is 4.5 to about 8.4 but can be lower than 3.5 and higher than 9.0. CEC is also dependent on the soil pH.

### 6.4 Soil Organic Matter (SOM)

The organic matter component of soil is comprised of substances from plant and animal decomposition, material synthesized by organisms, and cells/debris from soil organisms. SOM impacts the physical and chemical properties of soils, including soil quality and function.

### 6.5 Soil Formation and Horizons

**Figure 2.**
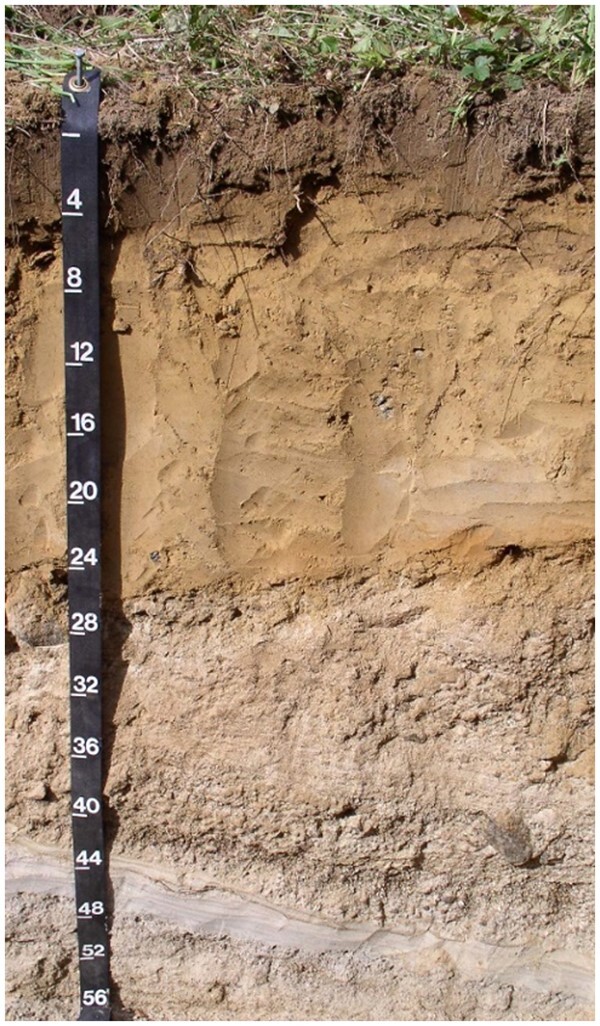
Soil profile with measurements in inches. Reprinted from https://www.nrcs.usda.gov/wps/portal/nrcs/detail/nj/soils/?cid=nrcs141p2_018867

Soils are formed by five main soil-forming factors: (1) climate, (2) parent material (bedrock and deposited sediments), (3) organisms (micro and macro), (4) relief (topography), and (5) time. Time as a forming factor refers to the time of active weathering versus the standard linear time scale. For example, a soil in Hawaii can be considered older than a soil found in North America due to active weathering. These five soil-forming factors lead to unique characteristics in each soil and the formation of soil horizons along a vertical profile (Figure 2). There are five identified horizons, called O, A, E, B, and C horizons layered above the unweathered parent material. Each horizon has distinct properties as defined in the Terms and Definitions. A soil may contain all or just a few of these horizons. The top horizon may be an O horizon of loose, partly decayed organic matter or an A horizon consisting of mineral matter mixed with organic material. For most experimental purposes related to the very upper portion of the Earth’s crust, the O, A, and B horizons tend to dominate sample collection and handling practices. Figure 2 is an actual soil profile showing the boundaries and variability of depth of horizons. The soil in Figure 2 has a 4-inch A horizon above a 20-inch B horizon. The C horizon is of an unknown depth. Soil horizons can vary in depth from a few inches to 40 feet or more.

## 7 Characterization and Selection of Soils

### 7.1 Soil Characterization Tests

#### 7.1.1 Physico-Chemical Characteristics

Soil is a very complex medium, and the physico-chemical characteristics of a soil can impact the performance of an analytical method or system. Three main soil properties are expected to have the most impact on an experiment or test: organic carbon content, clay content/soil texture, and soil pH. In addition, moisture content can affect the viability of biothreat agents as well as interactions between biothreat agents and chemical or biochemical constituents in the soil.

Methods for determining soil characteristics should be selected from standardized procedures, preferably the ISO methods listed in the *Handbook of Soil Analysis* (3). Most of these methods are performed at agricultural analytical laboratories across the nation (Table 1). When submitting a soil for analysis, it is recommended the laboratory’s soil preparation steps are utilized and the laboratory is alerted prior to sending the soil if it is not from the local region. Most analytical laboratories will calibrate instruments for soil characteristics specific to their region. If sending a soil from another part of the nation or the world, alerting them to the possible differences will allow them to tailor their methods towards the expected characteristics of the sample. For example, soil pH varies greatly with more acidic soil typically found on the east coast and more basic soils in the western United States. Alerting the laboratory that a soil may have a different pH than typically found in the local region will ensure they calibrate the pH probe correctly for the soil. Additional tests often offered by these analytical laboratories include CEC, moisture content, and water holding capacity. Samples to be submitted to a laboratory for chemical and physical characterization should be sterilized by autoclaving (*see* Section 8.6) with the understanding that this impacts culturing and assessment of heat labile materials. Alternatively, there are field test kits available for determination of soil moisture and other parameters.

**Table 1.** U.S. academic and nonacademic resources for soil information, testing, data, and archives

Organization	Website^a^	Comments
**Academic soil science departments**		
Michigan State U.	https://www.canr.msu.edu/psm/	Soil and plant nutrient laboratory
North Carolina State U. at Raleigh	https://cals.ncsu.edu/crop-and-soil-sciences/	Particle size and water retention analyses
North Dakota State U.	https://www.ndsu.edu/soils/	Soil testing laboratory
Ohio State U.	https://senr.osu.edu	Soil fertility laboratory
Oklahoma State U.	http://pss.okstate.edu	Soil testing laboratory
Pennsylvania State U.	https://ecosystems.psu.edu/graduateprograms/soil-science	Soil testing laboratoryhttps://agsci.psu. edu/aasl/soil-testing
Southern Illinois U. Carbondale	https://coas.siu.edu/academics/departments/plant- soil-agricultural-systems/index.html	
Texas Tech U.	http://www.depts.ttu.edu/pss/index.php	
U. of Arizona	https://swes.cals.arizona.edu/home	
U. of Delaware	https://canr.udel.edu/plsc/	Soil testing laboratory
U. of Florida	https://soils.ifas.ufl.edu	Soil testing laboratory
U. of Kentucky	http://pss.ca.uky.edu	Soil testing laboratory
U. of Maryland	https://enst.umd.edu	
U. of Wiconsin–Madison	https://soils.wisc.edu	
Utah State U.	https://psc.usu.edu	
**Nonacademic resources**		
National Ecological Observatory Network (NEON)	https://www.neonscience.org/data/neon- biorepository/archival-samples-catalog	Biological, genomic, and geological archival samples
International Soil Carbon Network	https://iscn.fluxdata.org/data/contribute-data/ soil-archive-survey/	Information gathering on archived soils
	https://iscn.fluxdata.org/resources/overview/	Directory of soil laboratories, methods, and other resources
USDA NRCS^b^	https://www.nrcs.usda.gov/wps/portal/nrcs/detail/soils/ contactus/?cid=nrcs142p2_053951	Directory of state soil scientists
	https://websoilsurvey.nrcs.usda.gov/app/	Web soil survey
Soil Science Society of America	https://www.soils.org	International scientific society
Critical Zone Exploration Network and Critical Zone Observatories	http://criticalzone.org/national/about/partner /critical-zone-exploration-network/	Compilation of datasets

a All websites accessed on April 15, 2019.

b USDA NRCS = U.S. Department of Agriculture Natural Resources Conservation Service.

#### 7.1.2 Microbiological Characterization

The microbiological stability of soil samples can be gauged by measuring RNA content over time. Although free RNA degrades rapidly, encapsulated RNA (e.g., RNA viruses) can be very stable. Additionally, many vegetative forms of bacterial pathogens persist for days to months in soil (4). A time-course study of pathogens in soil can be performed using RNA (or messenger RNA) analysis or by culture (if culturable).

If nucleic acid preservation is desired, a commercial preservation solution with a validated expiration date is recommended. The preservative should be evaluated before use for compatibility with the method or system under evaluation. Likewise, commercial nucleic acid extraction kits intended for use with soil are recommended. Appropriate commercial bacterial standards should be used.

Most naturally occurring microorganisms do not grow on common culture media (5). However, culturing microbes does provide an indication of the diversity and types of microbes occurring in the soil (6). Culturing can also be used to indicate whether a soil was adequately sterilized (7). A general-purpose solid culture medium for soil samples is R2A Agar, which is a low-nutrient agar formulated to promote growth of stressed or slow-growing bacterial cells. To culture soil bacteria using the R2A method:

A 10-fold dilution is created by suspending 3 g soil sample in 27 mL of 0.1% (by mass) peptone solution.

Vortex the soil suspension for 30 s, shake 25 times in a wide arc, and allow the tube to sit for about 5 min until the coarse particles have settled.

Spread-plate 0.1 mL of the supernatant solution onto an R2A agar plate.

It is recommended that serial 10-fold dilutions in 0.1% peptone be prepared and plated in order to obtain isolated bacterial colonies. Dilutions of 10^−6^ through 10^−8^ could produce a few plates with 20–200 isolated colonies for most soils. Incubate the plates at 20–28°C for 10 days. Fast growing bacteria will appear in about 2 days while slow growing bacterial and other microbial colonies do not appear until 8–10 days of incubation (8).

### 7.2 Criteria for Selecting Soils

Soil selection depends on the intended use of the method or system. If an instrument or method is being developed for deployment in a very specific region of the world, then soils specific to that region should be collected. For intended uses not specific to one region, Table 2 from the Organisation for Economic Co-operation and Development (OECD) Guideline 106 (9) provides soil characteristics covering a wide range of pH, organic content, and clay content typically found in temperate geographical zones. This international guideline was developed in 2000 and used by the U.S. Environmental Protection Agency (EPA) in studies concerning the mobility, distribution, and degradation of chemicals in soils. Ideally, all seven soil types from Table 2 should be included in the experimental testing. If it is not possible to test all seven types of soil, it is recommended to test at least five soils of varying characteristics following the guidance from Table 2. There may be cases in which extreme soil types (e.g., coastal soil with high salt content) are required for a specific purpose. In these cases, the soils of interest should be characterized and documented prior to testing, which may include additional characterization tests for specific parameters of interest. As much as possible, a variety of soils covering the range of the parameter of interest should be included in the experimental testing.

**Table 2.** Guidance for selection of soil samples^a^

Soil type	pH range (in 0.01 M CaCl_2_)	Organic carbon content, %	Clay content, %	Soil texture
1	4.5–5.5	1.0–2.0	65–80	Clay
2	>7.5	3.5–5.0	20–40	Clay loam
3	5.5–7.0	1.5–3.0	15–25	Silt loam
4	4.0–5.5	3.0–4.0	15–30	Loam
5	<4.0–6.0	<0.5–1.5	<10–15	Loamy sand
6	>7.0	<0.5–1.0	40–65	Clay loam/clay
7	<4.5	>10	<10	Sand/loamy sand

a From OECD Guideline 106 (9).

Site-specific soil testing may be required for a site survey, assessment of decontamination or remediation effectiveness, or validation of a method for assessment of decontamination and remediation efforts. In cases such as these, a target testing grid should be laid out over the test area and a soil sample from each grid area should be collected (*see* Section 8.4). For decontamination and remediation assessments, soil samples should be collected before, during, and after the controlled experimental release of agent or experimental decontamination treatment. Even on a small test grid, soil types and characteristics can vary. A soil survey or online tools like the web soil survey (Table 1) should be consulted to determine differing soil types and their expected locations. Documentation of soil characterization tests as well as experimental results from the site should be archived in a database for future reference.

## 8 Soil Sampling, Processing, and Type

### 8.1 Soil Sampling

#### 8.1.1 Tools and Supplies

- *Shovel, spade, or trowe*l.
- *Soil probe*.—Smooth hollow tube device for collecting a small diameter soil core.
- *Soil auger*.—Manual drill with hollow core (“bucket”) for collecting disturbed samples.
- *Sample containers*.—Resealable plastic bags, tubes with caps, or plastic buckets with lids, etc., of an appropriate size.
- *Knife*.—Straight blade, not pocket knife. Alternatively, scissors could be used.
- *Ice pick*.
- *Marker pen*.—For labeling sample containers.
- *Insulated box*.—Containing ice.

#### 8.1.2 Soil Collection

Sample containers can range from store-bought resealable bags to sterile plastic bags or screw-cap tubes as needed, depending on the required sample size. The size of soil samples collected should be determined based on the application. If planning to send the samples for soil characterization, at least one large (i.e., 1 kg) sample should be collected. Samples collected for other purposes could be as small as a few grams placed into a microcentrifuge tube.

For some applications, it may be desirable to avoid contaminating a soil with extraneous microbes. Aseptic sampling techniques can be challenging, however, to practice in the natural environment. Pre-autoclaved sampling tools and containers can be individually wrapped and brought to the field in a second container until needed in order to prevent contamination before using. Alternatively, metal sampling tools can be sterilized in the field by washing with water, rinsing with 95% ethanol and evaporating by flame (a household lighter is sufficient for this purpose). If sterility of tools and containers is not required, then clean tools and ordinary store-bought resealable bags are acceptable.

When using a soil probe to collect a core sample, push the probe down and then gently pull the probe back out keeping the probe vertical and have a collection bag ready to collect the core sample. When using an auger to collect a sample, make markings on the auger extension pole in increments equal to the length of the auger itself so the auger is not drilled beyond one auger depth before it is pulled up to collect the sample. Twist the auger into the soil until the first mark is at the top of the hole, gently pull the pole and auger up to the surface, keeping the auger vertical, and collect the sample from the interior of the auger bucket. Remove any excess soil and gently put the auger back into the hole. Drill the auger into the soil until the next mark on the pole is at the top of the hole and repeat the cycle until the desired depth or number of samples has been reached.

Commercial sample collection kits are also available for small and large sample sizes (e.g., Quick Silver Analytics, Hampstead, NC, USA, and AMS, Inc., American Falls, ID, USA).

### 8.2 Surface Sampling

For surface sampling, large samples (buckets) from wide ranging sites are generally collected. When collecting a soil from the surface, including the O and A horizon, first research the depth of the A horizon with the web soil survey (Table 1) and then visually confirm to prevent collecting the more clay-rich B horizon. To avoid collecting disturbed soils, select a site away from roads and man-made structures. Using a shovel or spade, collect soil down to about an inch above the A–B boundary layer in a circular area of desired diameter regularly confirming that only the A horizon, with some O if present, is collected. Place the collected soil from each site in a bucket or large bag. It is difficult to collect soil close to a tree or bush due to their root systems, but if microbial diversity is desired, collect plant roots along with the soil and place all samples on ice immediately after collection. If collecting soils on military ranges or near former military ranges, samplers should review their organization’s sampling and safety procedures. Unexploded ordinances could pose a significant hazard and a sweep of the area may be recommended prior to any soil sampling. Special shovels and trowels made from an aluminum bronze copper alloy can be used to prevent an incident if a hazard is found while sampling.

### 8.3 Profile Sampling

In this context, profile sampling relates to soil decontamination and remediation. Organisms or molecules of interest may percolate through the soil after a rain or snow melt event. Soil samples can be collected from the different horizons to test the extent of contamination and effectiveness of remediation. For sampling within each horizon, a soil auger ranging from 3 to 12 inches can be used. Approximate soil horizon depths can be researched using the web soil survey (Table 1) but should be confirmed in the field. Samples should be collected from roughly the middle of the horizon. For aseptic collection, a sterile spatula can be used to first remove a few centimeters of soil from the outer circumference of the soil sample that had contact with the auger during collection. A second sterile spatula can be used to collect the inner core sample from the center opening of the auger.

### 8.4 Sample Types and Practices

Prior to using a soil for detector or assay testing, it is important to consider experimental design and what is required to have defensible data prior to collecting a soil or starting an experiment.

Judgement samples are those resulting from an individual deliberately avoiding a particular area, thus exercising judgement in selecting sampling sites, and therefore are highly biased and not recommended for most purposes. Judgement samples should be reserved for instances when the individual collecting the sample is only using the sample as a source of soil microbes.

From a statistical perspective, simple random samples are more representative of an area than judgement samples as each sample has an equal opportunity to be selected. A common technique for collecting simple random samples is to establish a grid consisting of two sets of parallel lines at right angles to each other. Each line is assigned a unique number. Pairs of numbers are drawn from a random number table and used to establish intersecting points that denote where a sample will be collected. This process is repeated until the number of required samples is reached. Another simple random sample method is to use a pair of random numbers to designate a distance and angle from a selected starting point.

Stratified random sampling is similar to simple random sampling except that the area of interest is divided into smaller subareas. These subareas are selected based on known variations in the soil or other factors of interest. Samples are collected within each subarea in a random manner as described above. The advantage of stratified random sampling is that a researcher can compare results between the subareas and possibly correlate results to the factor of interest.

Systematic sampling is comprised of sampling at predetermined points or intervals, such as points along parallel lines or intervals based on the distance from a point. This type of nonrandom sampling is performed in order to ensure an area is well understood. Systematic sampling is useful for sampling after an outdoor activity with the sampling lines informed by the activity and its location.

Composite samples are those that are comprised of a number of smaller samples mixed together in order to reduce the cost of analyzing each sample individually. For example, random samples collected within a subarea can be composited and analyzed to produce a single result for that subarea. Once prepared, the composite sample can no longer provide any information on the variation among the individual samples. In order for results from composited samples to be valid, certain conditions must be met. First, all composite samples should be comprised of an equal number and mass of individual samples; second, there must be no interactions between the individual samples as these interactions could skew the results; and third, the study’s objectives must include obtaining an unbiased estimate of the mean (10).

Once the sample type is determined, it is recommended to follow sound sampling practices prior to using the soil sample. Parameters such as how much soil is required for measuring soil properties and for the experiment or testing could be calculated prior to collecting the sample. It is also recommended to consider how many samples or tests will be required to provide defensible results. Several websites provide guidance on sampling practices and experiment setup that could be considered during planning phases of using the soil samples (11–13). Considering sampling error prior to collecting and using soil could provide more sound results.

### 8.5 Soil Processing

Soil processing is experiment-dependent. When a soil is collected for analyses, the typical protocol is to air dry the soil for several days or place the sample in a drying oven at 105°C overnight. After removing rocks and plant debris, the dried sample is then crushed with a mortar and pestle and passed through an American Society for Testing and Materials (ASTM)-compliant 2 mm standard sieve to remove gravel. This protocol is not appropriate, however, for maintaining the microbiological integrity of the sample.

If a soil sample is collected in order to retain the soil fauna, then it is recommended that the soil be stored on ice immediately after collection and analyzed as soon as possible. Evidence suggests that drying of the soil or long-term storage, even at 4°C, can result in changes to the soil fauna (10). The soil moisture level should be measured at the time of collection as it may change during processing. The greatest concentration and diversity of soil organisms tends to be in the rhizosphere near plant roots. When collecting the soil, gently shake loose the soil from around the roots. The soil should not be dried but quickly passed through an ASTM-compliant 5 mm standard sieve, stored at 4°C, and used soon after or further processed by sterilization if appropriate. The microbiological composition of the soil sample will change over time due to drying, changes in oxygen levels, and competitive microflora.

In some cases, it may be desirable to perform an appropriate extraction of the soil at the time of collection and processing in order to preserve nucleic acids, proteins, or other potentially degradable molecules from the soil. The specific extraction procedure employed must be well understood chemically in order to understand the partitioning of molecules between the extraction solution and the insoluble soil components. Individual subsamples of soil material can be extracted with different extraction solutions in order to preserve multiple types of extracted components from the same soil sample. As mentioned in Section 7.1.2, commercial reagents and kits are available for preservation and extraction of biochemical components from soils.

### 8.6 Soil Sterilization Methods

For applications that require sterilized soil, autoclaving and gamma irradiation are the most common methods employed (14). Both methods have pros and cons that must be considered.

Autoclaving soil is an inexpensive and readily available method, but sterility cannot be guaranteed even after three autoclave cycles as spore-forming bacteria and other organisms may survive the procedure. Autoclave soils while still moist for three autoclave cycles with a period of 1–2 days between cycles. Autoclaving moist soil will encourage spore-forming bacteria to enter a vegetative state prior to the next autoclave cycle, however, it is not expected that the soil will be fully sterile. Soil minerals change when exposed to heat and pressure, so autoclaving is also expected to affect the minerology of the soil. Samples to be submitted to a laboratory for chemical and physical characterization should be sterilized with the understanding that this impacts culturing and assessment of heat-labile materials.

Gamma irradiation is a more expensive and less available method, but it is not expected to change the soil minerology and is able to inactivate spore-forming bacteria. However, gamma irradiation could decrease the organic matter content overall, so the organic content should be measured pre- and post-irradiation. Current procedures recommend using a ^60^Co or ^137^Cs source. Place up to 25 kg soil in either glass containers with a screw top lid or polyethylene bags for irradiation. If irradiating large amounts of soil, it is recommended that well-mixed soil be divided into smaller containers. The samples should be irradiated with 0.03 to 0.06 MGy or 3 to 6 Mrad (14).

### 8.7 Soil Dry Mass

Soil that has been dried on a laboratory bench still contains about 3–5% moisture depending on the humidity in the laboratory. Therefore, soil mass is reported as the oven-dry weight. Four replicate 5 g subsamples of the soil are placed into preweighed aluminum pans and weighed. The air-dry soil mass of each replicate is determined as the total air-dry mass (g) minus the pan mass (g) and recorded. The pans are placed in a drying oven (105–110°C) for at least 18 h and then allowed to cool in a desiccator containing calcium sulfate until the soil reaches room temperature. The pans containing subsamples are weighed again and the oven-dry soil mass is determined as the total oven-dry mass (g) minus the pan mass (g). The dry soil fraction is then calculated as:

Dry fraction=oven−dry soil mass/air−dry soil mass

### 8.8 Soil Storage

Once processed, soil samples should be subdivided and stored to preserve the physical, chemical, microbiological, and biochemical (protein and nucleic acid) characteristics and composition. Ideally, subsamples should be archived with associated collection, processing, and characterization metadata in a searchable database.

#### 8.8.1 Air-Dried Soils

 Soils that are processed by air drying and sieving can be stored at room temperature in a closed-lid container for up to 5 years. Before using the soil for experiments, the soil should be well mixed. Soils should also be recharacterized prior to experiments every 2 years.

#### 8.8.2 Soils for Microbiological and Biochemical Applications

Soils that are used specifically for their microbial community should be used either immediately after collection or as soon as possible after that time. Until usage, samples should be stored at 4°C in a sealed container to limit microbial activity without having a major impact on the microbial community composition. Studies show that storage for more than 21 days at 4°C begins to impact some of the measurable microbial activities (10).

### 8.9 Manufactured Soil

For some applications, a manufactured soil may be appropriate for preliminary studies. However, a manufactured soil is not recommended for full testing as it does not recapitulate all of the properties of real soil. Manufactured soils are prepared using defined recipes. As such, they do not include soil organisms that are an essential part of the soil. Microorganisms impact nutrient cycling, soil structure, water retention, and other processes that naturally occur in soil. The manufactured soil recipes provided here use only one clay mineral. There are over 2000 known minerals with 20 major minerals that can be found in soil.

All materials that will be used to prepare manufactured soil should be pretreated as follows: Wash in dish detergent and tap water; thoroughly rinse with hot tap water to remove soap; rinse four times with ASTM-compliant Type 2 purified water (>5 MΩ cm at 25°C; TOC <30 ppb); and rinse three times with ASTM-compliant Type 1 purified water (18 MΩ cm at 25°C; TOC <10 ppb). Additionally, all glassware and plasticware should be rinsed with 0.1 N nitric acid after the Type 2 water rinses and prior to the Type 1 water rinses.

It is recommended that two manufactured soils be prepared consisting of sand and montmorillonite clay with and without humus. The sand component should be purified silica sand (SiO_2_) obtained from a quarry, air-dried, and characterized. The montmorillonite clay and humus should be purchased and well characterized. Prior to use, the humus should be air-dried, passed through a sieve (≤5 mm), milled in a soil grinder, then passed through a second sieve (≤2 mm).

Prepare the two soil mixtures as follows: All masses are oven-dry masses. The textural class of both soil mixtures is expected to be Loamy Sand (15).

- *Manufactured Soil 1 (90% sand + 10% clay).—*In each of two 1 L acid-washed jars, place 450 g silica sand. To each jar, add 50 g montmorillonite clay. Tightly close each jar with a Teflon-lined lid and mix on a 3-dimensional soil mixer for at least 18 h.
- *Manufactured Soil 2 (88% sand + 8% clay + 4% humus).—*In each of two 1 L acid-washed jars, place 440 g silica sand. To each jar, add 40 g montmorillonite clay and 20 g humus. Tightly close each jar with a Teflon-lined lid and mix on a 3-dimensional soil mixer for at least 18 h.

## 9 Additional Soil Resources

### 9.1 Nonprofit, Laboratory, and Archival Resources

Table 1 lists available nonprofit resources for information, analytical testing, and/or archived soils. Included are active academic soil science departments at leading universities, governmental organizations, and nonprofit nongovernmental organizations. Nearly all of the universities listed have extension services for their communities and some include laboratory testing services as indicated. Table 1, however, is not intended to be comprehensive of all available resources.

### 9.2 Standard Reference Material Resource

The National Institute of Standards and Technology (NIST) is a source of Standard Reference Materials (SRMs), which are well-characterized materials that meet NIST-specific certification criteria and are issued with a certificate of analysis. Several soil, sludge, and sediment SRMs are currently available for purchase: https://www-s.nist.gov/srmors/detail.cfm?searchstring=soil (accessed April 15, 2019). Product listings are subject to change. Alternate commercial sources of SRMs may be available.

### 9.3 Shipping and Disposal Resources

Shipping regulations are in place to prevent the spread of invasive species. Regulations and permits are under the purview of the U.S. Department of Agriculture (USDA) Animal and Plant Health Inspection Service (APHIS). Visit the APHIS website for more information on shipping and receiving soil: https://www.aphis.usda.gov/aphis/ourfocus/planthealth/import-information/permits/regulated-organism-and-soil-permits/sa_soil/ct_regulated_organism_soil_permits_home (accessed April 15, 2019).

Additionally, the International Air Transport Association (IATA) publishes the Dangerous Goods Regulations for air cargo: https://www.iata.org/publications/dgr/Pages/index.aspx (accessed April 15, 2019). These regulations should be consulted before shipping soil by air.

Due to the possible presence of invasive species, even if a state does not have regulations or quarantine rules in place, it is recommended that excess soils and plant material are sterilized by the autoclave method before disposal in a landfill.

## 10 Experimental Setup Recommendations

This section provides guidance on using one or several soil matrixes either as a variable affecting biothreat agent detection in a laboratory experiment setup or during field experiments. The previous sections provide guidance on obtaining and processing the soil prior to these types of experiments. During the laboratory experiments and evaluations, multiple soil matrixes could be tested in order to evaluate a range of possible conditions that could impact detectors or assays. Field experiments are specific to understanding what could happen after a release and tend to focus on one soil matrix.

### 10.1 Laboratory Test and Evaluation Setup

The laboratory experimental setup for testing and evaluating a biothreat agent method or system will depend on the method or system being tested. General guidance includes testing a minimum of three replicates of each sample and including positive and negative controls. Laboratory experiments also need to include multiple soil types. It is recommended to test at least five different soils with the varying characteristics listed in Table 2. Online resources, such as the web soil survey (Table 1), provide tools to search for locations to collect the various soils with the desired approximate pH, clay content, and organic content. Academic institutions with soil science departments may also be useful in obtaining specific soils (Table 1).

Prior to experimentation using a soil matrix as a testing variable in a laboratory environment, pH, water content, water holding capacity, organic carbon content, texture, and CEC of each soil should be measured. Other characteristics that may affect the experiments should also be included. If it is important to start an experiment quickly after sample collection, then soil pH and water content should be measured immediately, and the remaining characteristics measured at a later time. Soil pH and dry weight should be monitored during the course of the experiment as these could affect the performance of a biothreat agent detection method or system. If a soil is too moist, the soil can be dried to a more acceptable moisture content by air drying with frequent mixing in a sterile environment until the moisture content is low enough. Drying a soil is not recommended unless the method or system being evaluated requires dryer soil. Avoid collecting soil samples after a weather event in order to avoid overly moist soil. To add moisture to a soil, sterilized water can be sprayed onto the soil while monitoring the increase in mass by weighing the soil while spraying. Once added, mix the soil well to ensure even distribution of the added moisture.

Soils should be kept in the dark and preferably in an incubator. The container lid should be either opened regularly to ensure a consistent atmosphere or placed ajar with sterile cloth over the container opening preventing contamination in a humidity-controlled incubator.

If planning a time series experiment, either a large batch experiment or sacrificial replicates can be used. During each sampled time point, the water content of the soil should be measured as well in order to report the data in soil dry weight.

### 10.2 Outdoor Testing Setup

Field testing designed to simulate a biothreat agent release should be carried out in multiple environments and under multiple seasonal conditions. Typically, open-air field testing is conducted using a truth box of ca. ½ km^2^ in area. Multiple ground truth instrumentation towers are placed within the box and used in conjunction with meteorological instrumentation placed nearby at heights ranging from 1–3 m. Ground truth data typically include aerodynamic particle size, concentration, and fluorescence. Samples of airborne particles are collected via impinger or dry filtration to confirm identity and viability of the biosimulant using standard microbiological methods such as RT-PCR and agar plate colony forming unit (CFU) enumeration, respectively. Disseminations of biosimulant challenges are made just upwind of the test bed using a variety of particle-generating methods designed to mimic relevant threat conditions. Tracking of the aerosol cloud is accomplished in real-time using Light Detection and Ranging (LIDAR) technologies. This provides cloud surface dimensions and translational movement data. Concentration data by LIDAR is limited to the reflected cloud surface. No material identity is derived through LIDAR. Internal concentrations and confirmation of identity is measured by point instrumentation located at the towers within the truth box as the cloud passes through the test area. Systems being evaluated are typically assessed for the time to detect, limit of detection, and ability to accurately identify a biothreat agent.

Care should be taken to execute testing under mild weather conditions to limit interference by soil particulates. However, an understanding of the soil content with respect to the bio-flora and inert mineral particulate matter is crucial to assessing the field performance of biological detectors and identification systems. There is always potential for native soil materials to mask the detector’s operational ability as they are often re-aerosolized by windy conditions or through ground traffic near the test location. Systems under evaluation may be disabled or induced into a false alarm state when the technology is incapable of differentiating natural biological background from the challenge materials.

Test site selection must factor in these details to ensure success. Understanding the soil contents, including its variation through the seasons, allows interpretation of performance data and false alarms. Collected test data can then be used to adjust detection algorithms to optimize sensitivity and selectivity.

## REVIEW AND APPROVAL

The guidelines were reviewed by SPADA and approved on October 15, 2019.

### Disclaimer

Certain commercial equipment, instruments, or materials are identified in this paper only to specify the experimental procedure adequately. Such identification is not intended to imply recommendation or endorsement by NIST, nor is it intended to imply that the materials or equipment identified are necessarily the best available for the purpose.

*DTRA Statement*: Approved for public release; distribution is unlimited.
